# Identification of a natural human serotype 3 parainfluenza virus

**DOI:** 10.1186/1743-422X-8-58

**Published:** 2011-02-09

**Authors:** Hui-Ting Yang, Qing Jiang, Xu Zhou, Mu-Qun Bai, Hong-Li Si, Xiao-Jing Wang, Yan Lu, Heng Zhao, Hong-Bin He, Cheng-Qiang He

**Affiliations:** 1 College of Life Science, Shandong Normal University, Jinan, 250014, China; 2 LanZhou Institute of Biological Products, Lanzhou, 730046, China; 3 Institute of Dairy Cattle Research, Shandong Academy of Agricultural Science, Jinan, 250100, China

## Abstract

Parainfluenza virus is an important pathogen threatening the health of animals and human, which brings human many kinds of disease, especially lower respiratory tract infection involving infants and young children. In order to control the virus, it is necessary to fully understand the molecular basis resulting in the genetic diversity of the virus. Homologous recombination is one of mechanisms for the rapid change of genetic diversity. However, as a negative-strand virus, it is unknown whether the recombination can naturally take place in human PIV. In this study, we isolated and identified a mosaic serotype 3 human PIV (HPIV3) from in China, and also provided several putative PIV mosaics from previous reports to reveal that the recombination can naturally occur in the virus. In addition, two swine PIV3 isolates transferred from cattle to pigs were found to have mosaic genomes. These results suggest that homologous recombination can promote the genetic diversity and potentially bring some novel biologic characteristics of HPIV.

## Introduction

Human parainfluenza virus (HPIV) is known to induce acute respiratory infections (ARI) including lower respiratory tract infection, which is a leading cause of morbidity and mortality in infants and young children [1,2]. Until now, four serotypes of parainfluenza virus (PIV) infecting human being have been found. Especially, human PIV (HPIV) 1, 2 and 3 are the second leading causative agents of pediatric hospitalizations due to respiratory disease following respiratory syncytial virus (RSV) [3]. It is important to know the mechanism resulting in genetic and antigenic diversity of HPIV for controlling the pathogen.

As one member of the *Respirovirus *ge nus of the family *Paramyxoviridae*, HPIV is an enveloped non-segmented negative single-stranded RNA virus [4]. RNA viruses usually exhibit genetic variation, which can be attributed to their high rate of mutation during their replication process and the large population size [5]. In addition, homologous recombination has been recognized increasingly as a potentially important means of generating and shaping genetic diversity in positive strand RNA virus [6]. In several other members of the *Paramyxoviridae *family, Newcastle disease virus (NDV) [7-11] and human respiratory syncytial virus [12], natural recombinants have been detected. Moreover, attenuated vaccines were found to be able to influence the evolution process of NDV through exchanging their genetic material with circulating virus [7,9,11]. For HPIV, it is unknown whether there is natural recombinant virus circulating in the field.

In this study, we isolated and identified a natural type 3 HPIV mosaic isolate LZ22/FJ455842 (with a mosaic N gene) to show that homologous recombination can occur in HPIV3. Additionally, three HPIV1 isolates (HT88/U01082, HT89a/U01083 and HT89c/U01085) were found to be deposited in previous report. Interestingly, we also found that there were the two Swine PIV3 recombinants with mosaic L protein were thought to be associated with an cross-species infection in previous report [13,14]. Collectively, these recombination events suggested that homologous recombination played a role in HPIV genetic diversity and rapid evolution.

## Results and Discussion

The sequence of LZ22 complete genome has been deposited in GenBank (Access Number, FJ455842). And the PIV3 complete genome sequence alignment dataset was analyzed employing RDP3 software package for scanning the recombinant sequence. And the isolate LZ22 was found to have greatly strong recombination signal: RDP, p-value < 10^-21^; GENECOVY, p-value < 10^-21^, Bootscan, p-value < 10^-20^, MaxChi p-value < 10^-3^; Chimaera p-value < 10^-7^; Siscan, p-value < 10^-8^, 3Seq, p-value < 10^-13^. And a breakpoint was located at position 485. Two strains GP and ZHYMgz01 were suggested as representatives of its putative parent lineages.

**Figure 1:**
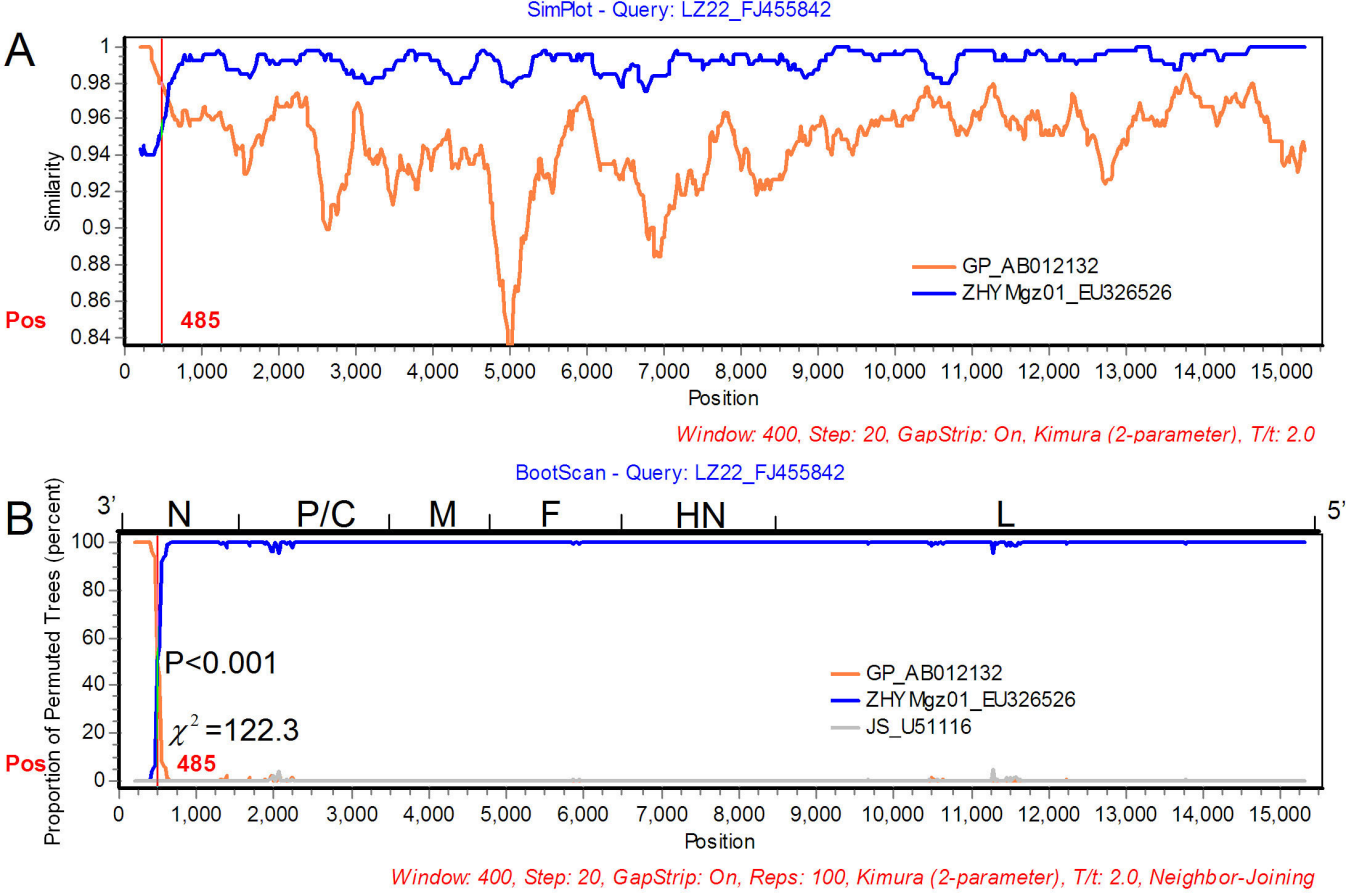
**(A, B) Results of Similarity and BootScanning analysis of the genome of LZ22_FJ455842**. The y-axis in Similarity plot (A) gives the percentage of sequence identity within a sliding window of 400 bp wide centered on the position plotted, with a step size between plots of 20 bp, while in BootScanning plot (B) represents the percentage of permuted trees. The χ2 of maximization and P value of Fisher's Exact test are shown near (or on) the vertical line. GP and ZHYMgz01 are used as two parental lineage sequences and JS_U51116 an outgroup sequence. The breakpoint is identified and located at position 485, with χ^2 ^value maximized. The query sequence LZ22_FJ455842 demonstrates greater sequence identity and BootScanning support with GP in the beginning region while otherwise with ZHYMgz01_EU326526 in the complementary regions.

And then, Simplot software package was used to determine the recombination event [15]. Employing the Findsites subprogram of SimPlot, one potential breakpoint was located at parsimonious regions with the maximization of χ^2^, from positions 485 to 615 (χ^2 ^= 122.3, *P *< 0.0001 of Fisher's exact test). A similarity plot (Figure 1A) which was constructed by using all sites, revealed that the sequence of LZ22 showed greater affinity with one putative parent lineage of GP in the region from position 1 to 485 than the other putative parent ZHYMgz01 (100% versus 94%). However, sequence from positions 486 to 15536, ZHYMgz01 shared greater similarity with LZ22 than GP (98% versus 95%). *P *value (Fisher's Exact Test) and χ^2 ^value of the breakpoint were shown on the vertical line in Figure 1. The identical evidence also appeared in BootScanning result (Figure 1B). The region from GP lineage spanned the amino terminal 1/3 of the N protein approximately.

**Figure 2:**
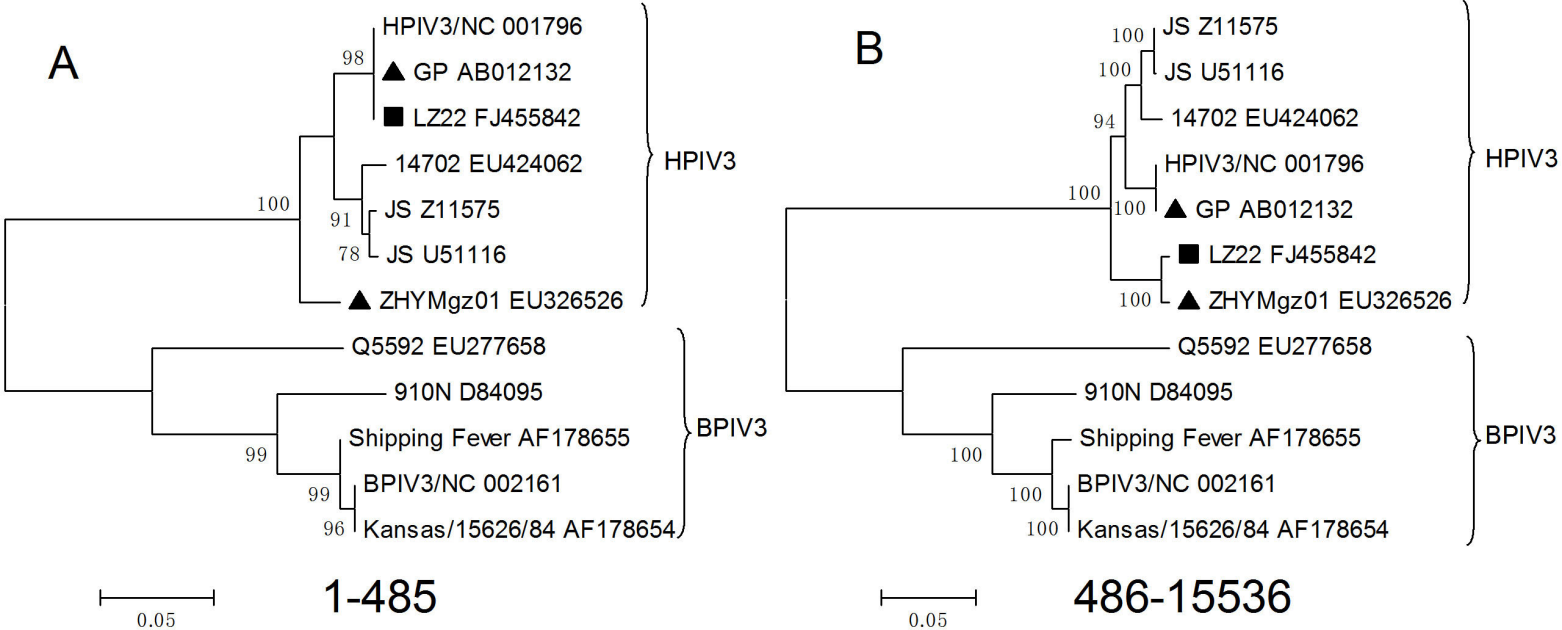
**Phylogenetic profiles of separate regions of LZ22_FJ455842 partitioned by cross-over events**. The scale corresponds to the number of nucleotide substitutions per site. The percentage of replicate trees in which the associated taxa clustered together in the bootstrap test (1000 replicates) is shown next to the branches (only N > 70% is shown). The putative recombinants were showed with "black square". The putative parent lineages were marked with "black triangle". (A) and (B) respectively represent the phylogeny of pre- (1-485) and post- (486-15536) part of complete segments delimited by the breakpoint. The pre-part of mosaics demonstrated higher level of congruence with the GP lineage, while the post-part converge with ZHYMgz01_EU326526.

At last, The phylogenic trees were also constructed using Mega 4 to determine the recombination events [16]. From positions 1 to 485, LZ22 and GP were clustered into the same sublineage with 98% bootstrap value, while ZHYMgz01 was grouped into distinct sublineage (Figure 2A). But, in the other portion, the arrangement of the phylogenetic tree reflecting the relationship of the three isolates was in contrast with the previous one (Figure 2B). The topology of the two phylogenetic trees around the breakpoint showed a significant statistic discrepancy when the mosaic was included in analyzed data (Shimodaira-Hasegawa test, P < 0.001), constituting a powerful evidence for recombination.

The minor putative parent of LZ22 was isolated from Japan, suggesting the existence of a global reservoir of HPIV with local subreservoirs supporting extensive levels of virus circulation, which permitted co-infection and resulted in the recombination event at last. The potential breakpoint 485 presents in gene N coding nucleoprotein spanning the region from positions 56 to 1737 of genomic sequence [17]. In the previous study, viable artificial chimeric HPIV3 recombinants were constructed, which contained the nucleoprotein open reading frame (ORF) from either BPIV3 Kansas or shipping fever (SF) strain in place of the HPIV3 N ORF. The artificial recombinant (PIV3) in which the nucleocapsid N protein had been replaced by that of bovine PIV3 was found to be attenuated in primates [18]. Here, we isolated a HPIV3 with a natural mosaic in NP ORF. Interestingly, before the breakpoint, the similarity of peptide sequences was up to 98.5% (129/131) although the similarity of gene sequences was only 94% between the two putative parent lineages. It is unknown whether the recombination in N gene is also associated with their adaptation in host cell via a changed virulence.

In addition, since there has been no report to show homologous recombination can take place between PIVs before this study; we analyzed 55 isolates (Table 1) from GenBank in order to determine to what degree genetic diversity of the virus is affected by the homologous recombination. Five additional mosaic PIV sequences were detected through the analysis of sequences from isolates characterized in previous reports:, HT88 (U01082) [19], HT89a [19] (U01083), HT89c (U01085) [19], 81-19252_Texas-81 (EU439429) [13,14] and 92-7783_ISU-92 (EU439428) [13,14] (Table 2). Two HN gene mosaics, HT88 and HT89a shared the same recombination event (Table 2), suggesting they descended from the same mosaic ancestor. Please also refer additional files 1, 2, 3, and 4 for the detail recombination information of each mosaic strain. These results suggested that homologous recombination did play a potential role in the evolution of the virus.

**Table 1:** Typical PIV isolates used in the study

GenBank Number	Strain	Serotype	Host	reference
NC_001796	unnamed	HPIV3	Human	[29]
AB012132	GP	HPIV3	Guinea pig	[30]
EU439429	81-19252_Texas-81	SPIV3	Swine	[13]
EU439428	92-7783_ISU-92	SPIV3	Swine	[13]
FJ455842	LZ22	HPIV3	Human	This study
NC_002161	unnamed	BPIV3	Bovine	[31]
EU424062	14702	HPIV3	Human	Unpublished
EU326526	ZHYMgz01	HPIV3	Human	Unpublished
D84095	910N	BPIV3	Bovine	[32]
AF178655	Shipping Fever	BPIV3	Bovine	[31]
AF178654	Kansas/15626/84	BPIV3	Bovine	[31]
Z11575	JS	HPIV3	Human	[33]
U51116	JS	HPIV3	Human	[34]
EU277658	Q5592	BPIV3	Bovine	[35]
U01082	HT88	HPIV1	Human	[19]
U01083	HT89a	HPIV1	Human	[19]
U01075	HT82a	HPIV1	Human	[19]
U70938	Mil-51/91	HPIV1	Human	[36]
U01074	HT81b	HPIV1	Human	[19]
U01073	HT81a	HPIV1	Human	[19]
U01076	HT82b	HPIV1	Human	[19]
U70937	Mil-49/91	HPIV1	Human	[36]
M86785	CH-B-73A	HPIV1	Human	[37]
U70947	Mil-63/91	HPIV1	Human	[36]
U70936	Mil-48/91	HPIV1	Human	[36]
M86786	CH-B-73B	HPIV1	Human	[37]
M86790	CH-B-83A	HPIV1	Human	[37]
U01079	HT85a	HPIV1	Human	[19]
M86791	CH-B-83B	HPIV1	Human	[37]
M86789	CH-B-79B	HPIV1	Human	[37]
U01084	HT89b	HPIV1	Human	[19]
U70948	Mil-64/91	HPIV1	Human	[36]
M86787	CH-B-77	HPIV1	Human	[37]
U01081	HT87	HPIV1	Human	[19]
M86784	CH-B-70	HPIV1	Human	[37]
U01080	HT85b	HPIV1	Human	[19]
U70943	Mil-58/91	HPIV1	Human	[36]
U01077	HT83a	HPIV1	Human	[19]
U70944	Mil-60/91	HPIV1	Human	[36]
U01085	HT89c	HPIV1	Human	[19]
U01078	HT83b	HPIV1	Human	[19]
AF016280	PIV1/Washington/ 20993/1964	HPIV1	Human	[38]
M86788	CH-B-79A	HPIV1	Human	[37]
U70939	Mil-52/91	HPIV1	Human	[36]
U70941	Mil-54/91	HPIV1	Human	[36]
M86781	CH-A-80	HPIV1	Human	[37]
U70946	Mil-62/91	HPIV1	Human	[36]
U70942	Mil-55/91	HPIV1	Human	[36]
U70945	Mil-61/91	HPIV1	Human	[36]
U70940	Mil-53/91	HPIV1	Human	[36]
M86783	CH-A-81B	HPIV1	Human	[37]
M86780	CH-A-66	HPIV1	Human	[37]
M86782	CH-A-81A	HPIV1	Human	[37]
M31228	unnamed	HPIV1	Human	[39]
M91648	C39	HPIV1	Human	[40]
X55803	unnamed	HPIV1	Human	[40]

**Table 2:** Characteristics of PIV intragenic recombinants

Strain	χ^2^_max_	Simplot identified breakpoints	RDP identified breakpoints	Putative parent lineages	Z-score	P-value*
LZ22	122.3	485-615	485	GP;	9.24	2.3E-9
				ZHYMgz01		
81-19252_Texas-81	366.9	8686-712	8688	910N;	9.34	2.5E-42
	83.7	12613-730	12595	Shipping_Fever		
	69.1	13619-36	13619		6.45	3.5E-6
	62.6	14134-245	14175			
92-7783_ISU-92	179.7	14134-206	14137	910N;	9.52	1.3E-7
	22.6	14863-5071	14989	Shipping_Fever		
HT88**	7.9; 8.5	300-396	392; 839	Mil-49/91;	6.07	1.2E-7
		828-867		HT89b		
HT89a**	7.9; 8.5	300-396	392; 839	Mil-49/91;	6.07	1.2E-7
		828-867		HT89b		
HT89c**	6.4; 14.2	351-392	351; 767	Mil-58/91;	5.98	3.8E-6
		534-828		Mil-51/91		

*Z-score and p values were calculated with the highest P = 0.01 by using SiScan program in RDP [28]

**HN genes of HT88, HT89a and HT89c were only analyzed. The positions of breakpoints indicated the relative sites in HN gene.

Interestingly, two mosaic viruses 81-19252_Texas-81 (U439429) and 92-7783_ISU-92 (EU439428) were reported to be involved in a cross infection between swine and bovine [13,14]. Both of the isolates had a mosaic L gene. The transcription and replication functions of the parainfluenza virus are associated with the large RNA polymerase protein. Additionally, polyadenylation, and RNA editing activities have to do with L protein [20]. The two putative mosaics were isolated from pigs in the United States [13,14] while both of their putative parent lineages (Shipping fever and 910N lineages) belonged to BPIV3. Viruses are largely species-specific with respect to their host and usually do not cross species boundaries [5]. Recombination processes will allow some viruses to acquire many of the key adaptive mutations in a single step, and thus make a major leap in fitness, which might result in a change of host tropism [21]. It might be necessary to further study whether the recombination event is relative to the BPIV3 cross-species infection.

In conclusion, this study provides the potential evidence that there is mosaic PIV in the field. Our observations show that homologous recombination is a molecular mechanism of PIV genetic diversity and evolution. Therefore, this study might be important for knowing the genetic basis resulting in the rapid change of PIV biologic characteristics.

## Material and methods

### Virus and sequencing

The virus LZ22 was isolated from lower respiratory tract of a patient infant with pneumonia in Lanzhou, Gansu Province of China in 2003. The virus was identified using previously described protocols [22]. After isolated, LZ22 was also purified 3 times by the plaque forming method in Vero cells. Before sequencing, LZ22 was passaged 13 times and amplified in Vero cells for RT-PCR. Viral RNA was extracted from Vero cell virus cultures using RNAeasy mini kit (Qiagen, Netherlands) following the manufacturer's instructions. Reverse transcription was performed using SuperScriptΠ one-step RT-PCR platinum Taq HiFi kit (Invitrogen, USA). 3' and 5' RACE were performed using 5'-full RACE CORE kit and 3'-RACE kit (Takala Dalian) to analyze the 3' and 5' UTR sequences. The primers of PCR and 3' and 5' RACE used in this study were listed in Table 3. The full genome of LZ22 was amplified and sequenced referring to previous report [22]. All PCR products were cloned into pGEM-T-vector (Promega USA) and sequenced by Takara Biotechnology (Dalian, China).

**Table 3:** Primers for sequencing of genome of HPIV-3 LZ22 strain

Fragment (Positions)	Primer (5'-3')	Sequence
NP	NPS	ACCAAACAAGAGAAGAGACTTGTTTGG
(1-1843)	NPA	TTCCTCTTCCCAAGAATCCATGATTTG
PP	PPS	GGACGAAATAGACGATCTGTTCAATGC
(1616-3485)	PPA	CTGTTCATTGACTTTGAGTGGTAATGG
M	MS	TCACTAGTTGCAGTCATCAACAACAGC
(3452-5183)	MA	CCCTTTGGGACTATTGACCAATACACC
F	FS	TGCAATTTTCCAACCTTCTTTACCTGG
4724-7104)	FA	AAGAAGCCTTGTATTCACTCCTGACTG
HN	HNS	AAATCGAGTGGATCAAAATGATAAGCC
(6644-8744)	HNA	TGTGTAATTGTGCTATTCTACCTTTAACG
L1	L1S	TGTTCAAAACAGAGATTCCAAAAAGCTGC
(8489-10684)	L1A	TCCAAATAGAGCCGTTGATTCATATCTCC
L2	L2S	CTGGAGATATGAATCAACGGCTCTATTTG
(10654-12958)	L2A	AATTGCATGTATAATGTCAGTATCATCCC
L3	L3S	TATTGGGATGATACTGACATTATACATGC
(12926-15461)	L3A	ACCAAACAAGAGAAGAACTCTGCTTGGTA
3'RACE (617)	S	TTGAACATAGAGCACAGACTGG
	A*	TACCGTCGTTCCACTAGTGATTT
(399)	Sn	GCTGATACGGATTCAGATTCATTCAAATTATC
	An*	CGCGGATCCTCCACTAGTGATTTCACTATAGG
5'RACE	S*	CATGGCTACATGCTGACAGCCTA
(15088)	A	AATAGCTCCTAAACATGATGGATACCC
	Sn	CGCGGATCCACAGCCTACTGATGATCAGTCGATG
(15423)	An	TGACATCTGCATTACTTCCATTTGTTGTTAGG

*Primers were supplied by manufacturer

### Recombination analysis

Compete genome and HN gene of PIV were retrieved from GenBank and aligned with CLUSTALW [23]. PIV3 compete genome and HPIV1 HN genes sequences analyzed in the study were listed in Table 1. Phylogenetic Neighbor-Joining (NJ) trees were set up by MEGA4 [16]. The nucleotide substitution models were optimized for Maximum-Likelihood (ML) trees employing jmodeltest (version 0.1.1) [24]. ML trees were constructed employing Phyml software with nucleotide substitution model of general time reversible model (GTR) and gamma distributed 4 (G4) [25], and displayed as graphics by using MEGA4 to determine the topology of each tree. Identification methods of homologous recombination were described as previous report [26,27]. Briefly, the sequence alignment files were sought for potential mosaic isolates using RDP software package [24,28]. The gene sequence similarity of mosaics and their putative parents were compared and displayed as graphics with Simplot software [15]. At last, incongruent phylogenetic relations of different gene regions delimited by crossover point were determined by phylogenetic trees.

## Competing interests

The authors declare that they have no competing interests.

## Authors' contributions

YHT, and JQ carried out sequence collection, alignment and recombination analysis and drafted the manuscript. ZX and BMQ provided LZ22 viral sequence information. SHL participated in sequence collection. WXJ, LY, ZH participated in revision of manuscript. HHB participated in its design and coordination. And HCQ designed the study and wrote the manuscript. All authors read and approved the final manuscript.

## Supplementary Material

Additional file 1 **The detail recombination information of mosaic strain 81-19252_Texas-81_EU439429**. (A, B) Results of Similarity and Bootscanning analysis of 81-19252_Texas-81_EU439429. The y-axis in Similarity plot (A) gives the percentage of identity within a sliding window of 500 bp wide centered on the position plotted, with a step size between plots of 20 bp, while in Bootscanning plot (B) represents the percentage of permuted trees. Shipping_Fever_AF178655 and 910N_D84095 were used as two parental sequences and Q5592_EU277658 an outgroup sequence. Four breakpoints were identified and located by GARD at position 8688, 12595,13619 and 14175, respectively, with value maximized. The query sequence 81-19252_Texas-81_EU439429 demonstrated greater sequence identity and Bootscanning support with 910N_D84095 in the second and fourth regions while otherwise with Q5592_EU277658 in the complementary regions. (C-G) Neighbor-Jointing Phylogenetic profiles of separate regions of 81-19252_Texas-81_EU439429 partitioned by cross-over events. The scale corresponds to the number of nucleotide substitutions per site. The putative recombinants were showed with "black square". C-G) represent the phylogeny of fir-(1-8688), sec-(8689-12595), thi-(12596-13619), fou-(13620-14175) and fin-(14176-15536) part of full length segment, respectively. The sec-and fou-part of mosaics demonstrated higher level of congruence with the 910N_D84095 lineage, while the otherwise converge with Shipping_Fever_AF178655. Click here for file

Additional file 2 **The detail recombination information of mosaic strain 92-7783_ISU-92_EU439428**. (A, B) Results of Similarity and Bootscanning analysis of 92-7783_ISU-92_EU439428. The y-axis in Similarity plot (A) gives the percentage of identity within a sliding window of 500 bp wide centered on the position plotted, with a step size between plots of 20 bp, while in Bootscanning plot (B) represents the percentage of permuted trees. Shipping_Fever_AF178655 and 910N_D84095 were used as two parental sequences and Q5592_EU277658 an outgroup sequence. Two breakpoints were identified and located by GARD at position 14137 and 14989, respectively, with value maximized. The query sequence 92-7783_ISU-92_EU439428 demonstrated greater sequence identity and Bootscanning support with Shipping_Fever_AF178655 in the middle region while otherwise with Q5592_EU277658 in the complementary regions. (C-E) Neighbor-Jointing Phylogenetic profiles of separate regions of 92-7783_ISU-92_EU439428 partitioned by cross-over events. The scale corresponds to the number of nucleotide substitutions per site. The putative recombinants were showed with "black square". C-E) represent the phylogeny of pre-(1-14137), mid-(14138-14989) and post-(14990-15536) part of full length segment, respectively. The pre-and post-part of mosaics demonstrated higher level of congruence with the 910N_D84095 lineage, while the mid-part converge with Shipping_Fever_AF178655. Click here for file

Additional file 3 **The detail recombination information of mosaic strains HT88_U01082 and HT89a_U01083**. (A, B, C, D) Results of Similarity and Bootscanning analysis of HT88_U01082 and HT89a_U01083. The y-axis in Similarity plot (A) gives the percentage of identity within sliding windows of 400 or 300 bp wide centered on the position plotted, with a step size between plots of 20 bp, while in Bootscanning plot (B, D) represents the percentage of permuted trees. Mil-49/91_U70937 and HT89b_U01084 were used as two parental sequences and CH-A-81A_M86782 and CH-A-81B_M86783 outgroup sequences. Two breakpoints were identified and located by GARD at position 392 and 839, respectively, with value maximized. The query sequences HT88_U01082 and HT89a_U01083 demonstrated greater sequence identity and Bootscanning support with HT89b_U01084 in the middle region while otherwise with Mil-49/91_U70937 in the complementary regions. (C-E) Maximum-Likelihood Phylogenetic profiles of separate regions of HT88_U01082 and HT89a_U01083 partitioned by cross-over events. The scale corresponds to the number of nucleotide substitutions per site. The putative recombinants were showed with "black square" (HT88_U01082) and "black triangle" (HT89a_U01083). E-G) represent the phylogeny of pre-(1-392), mid-(393-839) and post-(840-1728) part of complete segment HN, respectively. The pre-and post-part of mosaics demonstrated higher level of congruence with the HT89b_U01084 lineage, while the mid-part converge with Mil-49/91_U70937. Click here for file

Additional file 4 **The detail recombination information of mosaic strain HT89c_U01085**. (A, B,) Results of Similarity and Bootscanning analysis of HT89c_U01085. The y-axis in Similarity plot (A) gives the percentage of identity within sliding windows of 400 bp wide centered on the position plotted, with a step size between plots of 20 bp, while in Bootscanning plot (B) represents the percentage of permuted trees. Mil-58/91_U70943 and Mil-51/91_U70938 were used as two parental sequence and CH-A-81A_M86782 outgroup sequence. Two breakpoints were identified and located by GARD at position 351 and 767, respectively, with value maximized. The query sequence HT89c_U01085 demonstrated greater sequence identity and Bootscanning support with Mil-51/91_U70938 in the middle region while otherwise with Mil-58/91_U70943 in the complementary regions. (C-E) Maximum-Likelihood Phylogenetic profiles of separate regions of HT89c_U01085 partitioned by cross-over events. The scale corresponds to the number of nucleotide substitutions per site. The putative recombinants were showed with "black square". C-E) represent the phylogeny of pre-(1-351), mid-(352-767) and post-(768-1728) part of complete segment HN, respectively. The pre-and post-part of mosaics demonstrated higher level of congruence with the Mil-58/91_U70943 lineage, while the mid-part converges with Mil-51/91_U70938. Click here for file
