# Supplementary figures and images for: Identification of a natural human serotype 3 parainfluenza virus
Source: Virol J. 2011 Feb 9;8:58. doi: 10.1186/1743-422X-8-58 (PMC3045893; doi:10.1186/1743-422X-8-58)

Additional File 1


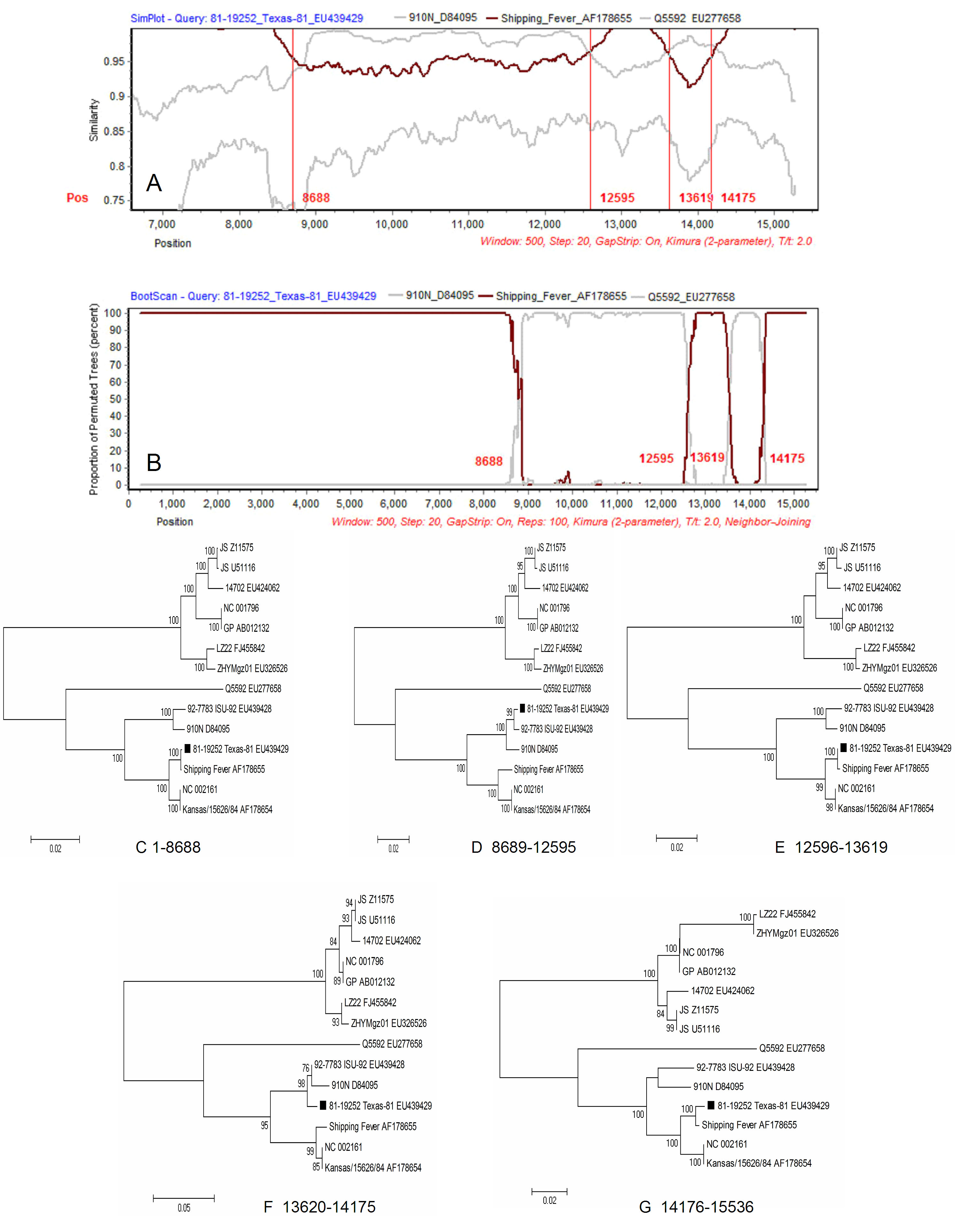

Supplement: Additional file 1 — The detail recombination information of mosaic strain 81-19252_Texas-81_EU439429. (A, B) Results of Similarity and Bootscanning analysis of 81-19252_Texas-81_EU439429. The y-axis in Similarity plot (A) gives the percentage of identity within a sliding window of 500 bp wide centered on the position plotted, with a step size between plots of 20 bp, while in Bootscanning plot (B) represents the percentage of permuted trees. Shipping_Fever_AF178655 and 910N_D84095 were used as two parental sequences and Q5592_EU277658 an outgroup sequence. Four breakpoints were identified and located by GARD at position 8688, 12595,13619 and 14175, respectively, with value maximized. The query sequence 81-19252_Texas-81_EU439429 demonstrated greater sequence identity and Bootscanning support with 910N_D84095 in the second and fourth regions while otherwise with Q5592_EU277658 in the complementary regions. (C-G) Neighbor-Jointing Phylogenetic profiles of separate regions of 81-19252_Texas-81_EU439429 partitioned by cross-over events. The scale corresponds to the number of nucleotide substitutions per site. The putative recombinants were showed with "black square". C-G) represent the phylogeny of fir-(1-8688), sec-(8689-12595), thi-(12596-13619), fou-(13620-14175) and fin-(14176-15536) part of full length segment, respectively. The sec-and fou-part of mosaics demonstrated higher level of congruence with the 910N_D84095 lineage, while the otherwise converge with Shipping_Fever_AF178655. [file 1743-422X-8-58-S1.DOC]

Additional file 2


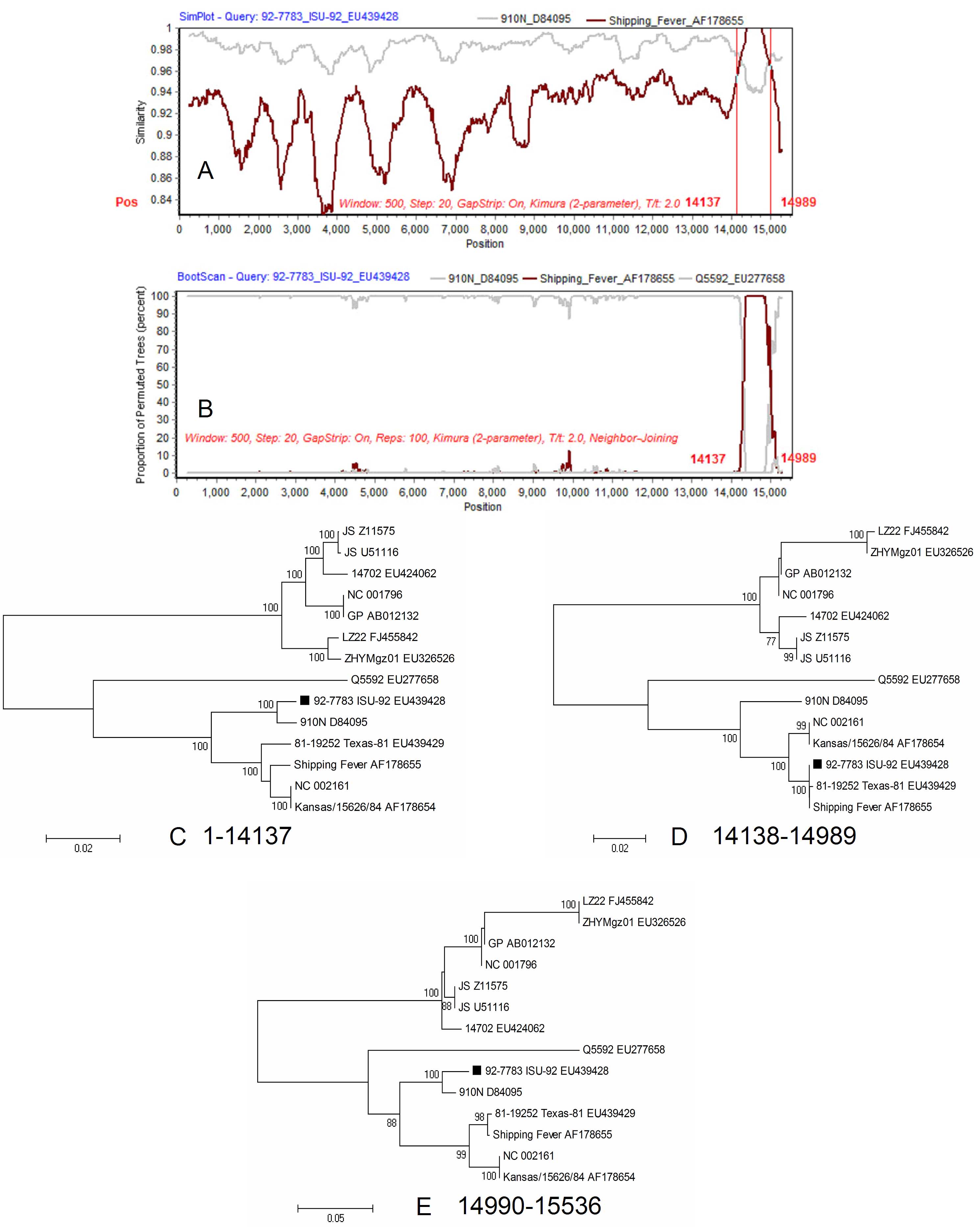

Supplement: Additional file 2 — The detail recombination information of mosaic strain 92-7783_ISU-92_EU439428. (A, B) Results of Similarity and Bootscanning analysis of 92-7783_ISU-92_EU439428. The y-axis in Similarity plot (A) gives the percentage of identity within a sliding window of 500 bp wide centered on the position plotted, with a step size between plots of 20 bp, while in Bootscanning plot (B) represents the percentage of permuted trees. Shipping_Fever_AF178655 and 910N_D84095 were used as two parental sequences and Q5592_EU277658 an outgroup sequence. Two breakpoints were identified and located by GARD at position 14137 and 14989, respectively, with value maximized. The query sequence 92-7783_ISU-92_EU439428 demonstrated greater sequence identity and Bootscanning support with Shipping_Fever_AF178655 in the middle region while otherwise with Q5592_EU277658 in the complementary regions. (C-E) Neighbor-Jointing Phylogenetic profiles of separate regions of 92-7783_ISU-92_EU439428 partitioned by cross-over events. The scale corresponds to the number of nucleotide substitutions per site. The putative recombinants were showed with "black square". C-E) represent the phylogeny of pre-(1-14137), mid-(14138-14989) and post-(14990-15536) part of full length segment, respectively. The pre-and post-part of mosaics demonstrated higher level of congruence with the 910N_D84095 lineage, while the mid-part converge with Shipping_Fever_AF178655. [file 1743-422X-8-58-S2.DOC]

Additional file 4


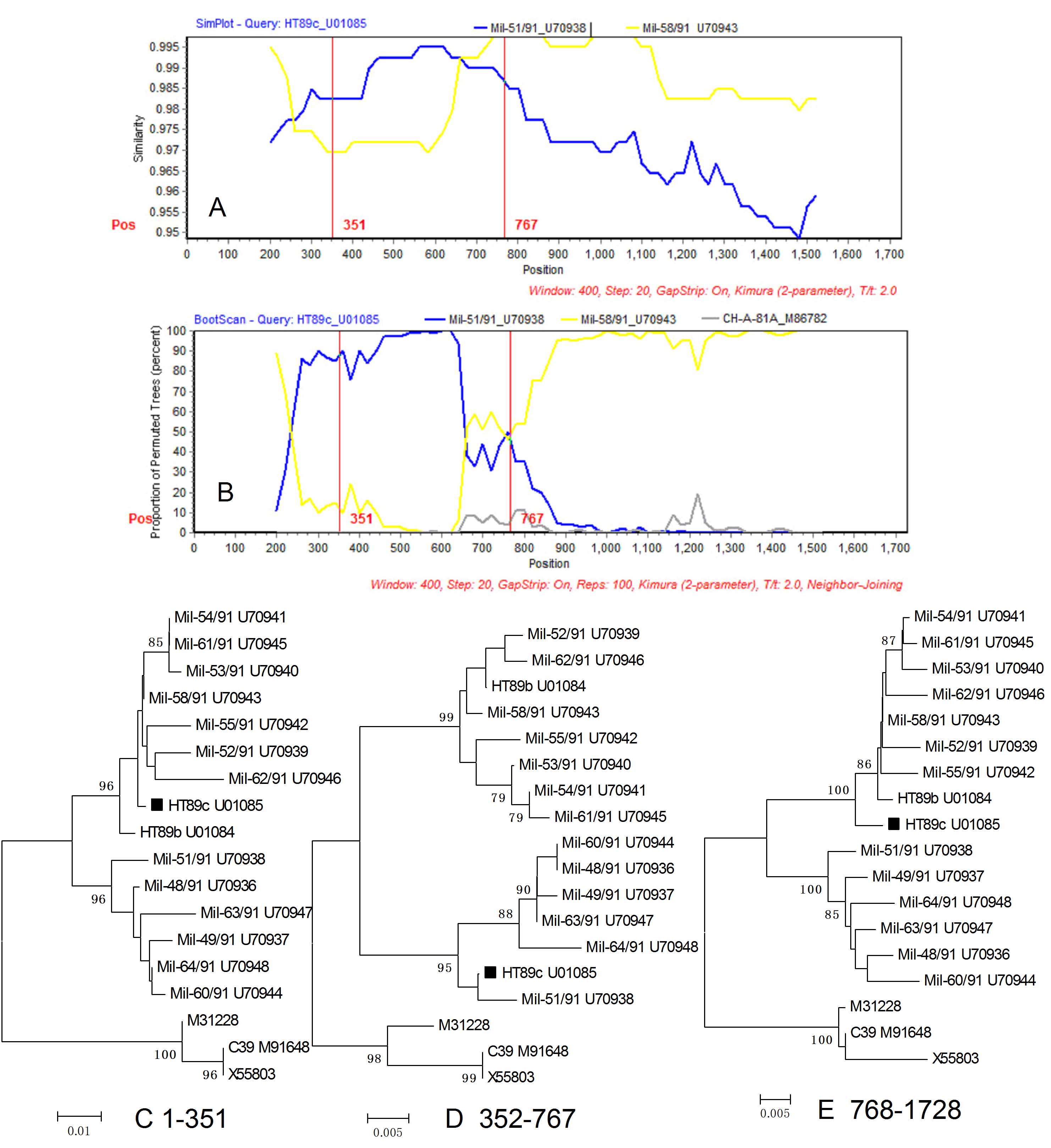

Supplement: Additional file 4 — The detail recombination information of mosaic strain HT89c_U01085. (A, B,) Results of Similarity and Bootscanning analysis of HT89c_U01085. The y-axis in Similarity plot (A) gives the percentage of identity within sliding windows of 400 bp wide centered on the position plotted, with a step size between plots of 20 bp, while in Bootscanning plot (B) represents the percentage of permuted trees. Mil-58/91_U70943 and Mil-51/91_U70938 were used as two parental sequence and CH-A-81A_M86782 outgroup sequence. Two breakpoints were identified and located by GARD at position 351 and 767, respectively, with value maximized. The query sequence HT89c_U01085 demonstrated greater sequence identity and Bootscanning support with Mil-51/91_U70938 in the middle region while otherwise with Mil-58/91_U70943 in the complementary regions. (C-E) Maximum-Likelihood Phylogenetic profiles of separate regions of HT89c_U01085 partitioned by cross-over events. The scale corresponds to the number of nucleotide substitutions per site. The putative recombinants were showed with "black square". C-E) represent the phylogeny of pre-(1-351), mid-(352-767) and post-(768-1728) part of complete segment HN, respectively. The pre-and post-part of mosaics demonstrated higher level of congruence with the Mil-58/91_U70943 lineage, while the mid-part converges with Mil-51/91_U70938. [file 1743-422X-8-58-S4.DOC]
