# Supplementary material for: Identification of a natural human serotype 3 parainfluenza virus
Source: Virol J. 2011 Feb 9;8:58. doi: 10.1186/1743-422X-8-58 (PMC3045893; doi:10.1186/1743-422X-8-58)

**Additional file 3**

Title: The detail recombination information of mosaic strains HT88_U01082 and HT89a_U01083.

Description: (A, B, C, D) Results of Similarity and Bootscanning analysis of HT88_U01082 and HT89a_U01083. The y-axis in Similarity plot (A) gives the percentage of identity within sliding windows of 400 or 300 bp wide centered on the position plotted, with a step size between plots of 20 bp, while in Bootscanning plot (B,D) represents the percentage of permuted trees. Mil-49/91_U70937 and HT89b_U01084 were used as two parental sequences and CH-A-81A_M86782 and CH-A-81B_M86783 outgroup sequences. Two breakpoints were identified and located by GARD at position 392 and 839, respectively, with value maximized. The query sequences HT88_U01082 and HT89a_U01083 demonstrated greater sequence identity and Bootscanning support with HT89b_U01084 in the middle region while otherwise with Mil-49/91_U70937 in the complementary regions. (C–E) Maximum-Likelihood Phylogenetic profiles of separate regions of HT88_U01082 and HT89a_U01083 partitioned by cross-over events. The scale corresponds to the number of nucleotide substitutions per site. The putative recombinants were showed with “black square” (HT88_U01082) and “black triangle” (HT89a_U01083). E–G) represent the phylogeny of pre-(1–392), mid-(393–839) and post-(840–1728) part of complete segment HN, respectively. The pre-and post-part of mosaics demonstrated higher level of congruence with the HT89b_U01084 lineage, while the mid-part converge with Mil-49/91_U70937.


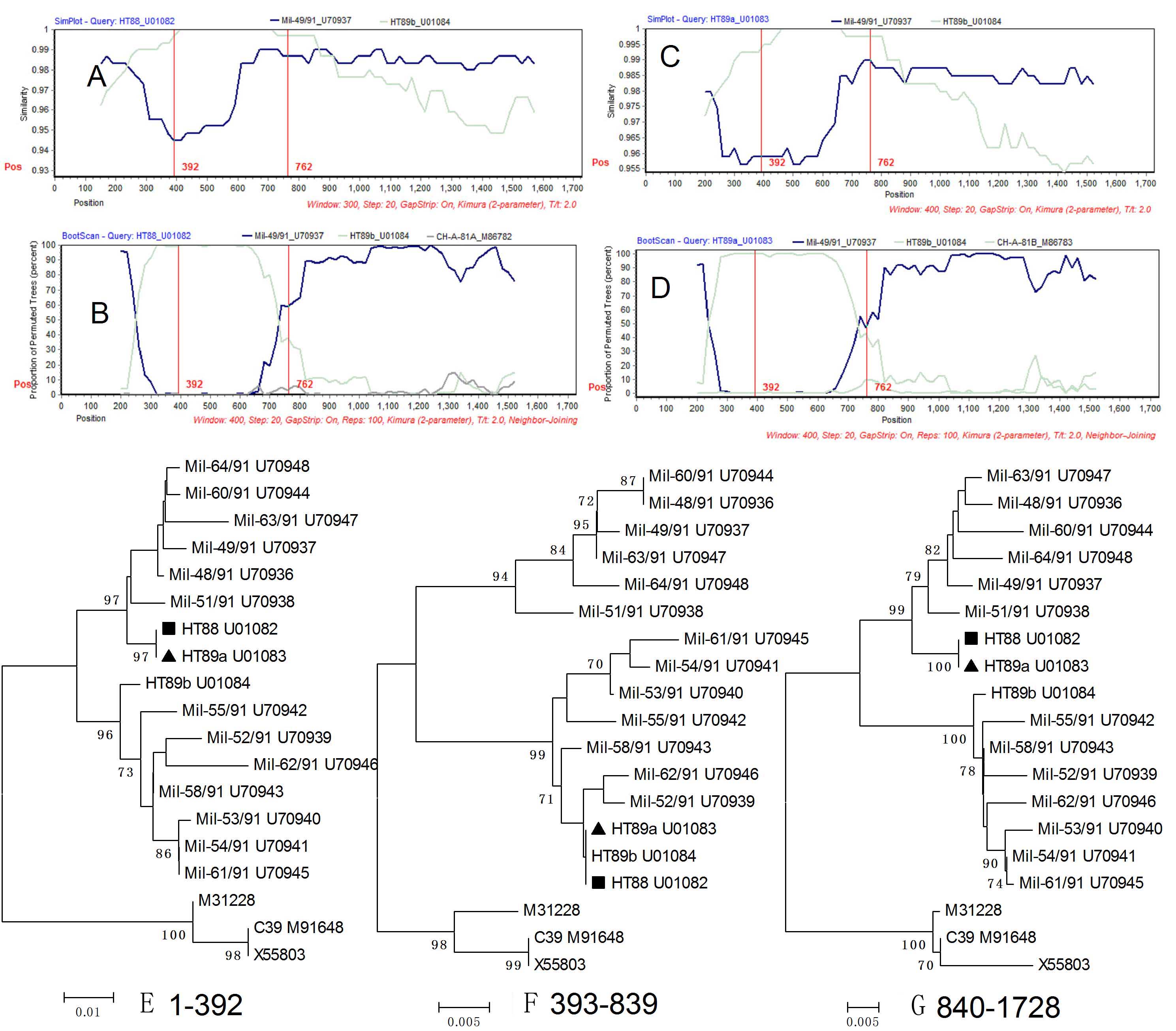

Supplement: Additional file 3 — The detail recombination information of mosaic strains HT88_U01082 and HT89a_U01083. (A, B, C, D) Results of Similarity and Bootscanning analysis of HT88_U01082 and HT89a_U01083. The y-axis in Similarity plot (A) gives the percentage of identity within sliding windows of 400 or 300 bp wide centered on the position plotted, with a step size between plots of 20 bp, while in Bootscanning plot (B, D) represents the percentage of permuted trees. Mil-49/91_U70937 and HT89b_U01084 were used as two parental sequences and CH-A-81A_M86782 and CH-A-81B_M86783 outgroup sequences. Two breakpoints were identified and located by GARD at position 392 and 839, respectively, with value maximized. The query sequences HT88_U01082 and HT89a_U01083 demonstrated greater sequence identity and Bootscanning support with HT89b_U01084 in the middle region while otherwise with Mil-49/91_U70937 in the complementary regions. (C-E) Maximum-Likelihood Phylogenetic profiles of separate regions of HT88_U01082 and HT89a_U01083 partitioned by cross-over events. The scale corresponds to the number of nucleotide substitutions per site. The putative recombinants were showed with "black square" (HT88_U01082) and "black triangle" (HT89a_U01083). E-G) represent the phylogeny of pre-(1-392), mid-(393-839) and post-(840-1728) part of complete segment HN, respectively. The pre-and post-part of mosaics demonstrated higher level of congruence with the HT89b_U01084 lineage, while the mid-part converge with Mil-49/91_U70937. [file 1743-422X-8-58-S3.DOC]
